# Insights into Sex and Gender Differences in Brain and Psychopathologies Using Big Data

**DOI:** 10.3390/life13081676

**Published:** 2023-08-02

**Authors:** Aura Zelco, Pattama Wapeesittipan, Anagha Joshi

**Affiliations:** Department of Clinical Science, Computational Biology Unit, University of Bergen, 5020 Bergen, Norway; aura.zelco@uib.no (A.Z.); pattama.wapeesittipan@uib.no (P.W.)

**Keywords:** sex and gender, psychopathology, neurodegenerative disorders, genomics, bioinformatics, imaging

## Abstract

The societal implication of sex and gender (SG) differences in brain are profound, as they influence brain development, behavior, and importantly, the presentation, prevalence, and therapeutic response to diseases. Technological advances have enabled speed up identification and characterization of SG differences during development and in psychopathologies. The main aim of this review is to elaborate on new technological advancements, such as genomics, imaging, and emerging biobanks, coupled with bioinformatics analyses of data generated from these technologies have facilitated the identification and characterization of SG differences in the human brain through development and psychopathologies. First, a brief explanation of SG concepts is provided, along with a developmental and evolutionary context. We then describe physiological SG differences in brain activity and function, and in psychopathologies identified through imaging techniques. We further provide an overview of insights into SG differences using genomics, specifically taking advantage of large cohorts and biobanks. We finally emphasize how bioinformatics analyses of big data generated by emerging technologies provides new opportunities to reduce SG disparities in health outcomes, including major challenges.

Though women make up for the half of the world population, they are severely underrepresented in preclinical and clinical research, including psychiatric diseases. This has severe consequences for female health. For example, from 1999 to 2000, of the 10 prescribed drugs withdrawn from the market by the US FDA, 8 posed higher risk in women than men [1]. Therefore, we urgently need more data and models to study sex differences in order to understand female neurobiology, behavior, and disease vulnerabilities. We will firstly define sex and gender terms and provide an evolutionary and developmental perspective.

## 1. Sex and Gender: Concepts, Developmental and Evolutionary Perspective

In most mammals, sex is studied and represented in binary form. Furthermore, in humans, sex is largely assumed binary—either male or female—with everything else being described as intersex. Intersex or disorders of sex development (DSD) are conditions where chromosomes or gonadal or anatomic sex development is abnormal [2]. The sex identity comes from chromosomes (XX for females, XY for males), gonads, and anatomy, and though they mostly function in harmony, there might be discordance between them. For example, chimerism is a rare state where a single individual contains cells from more than one genetic cell line. While most of these cases are detected with DSD symptoms, many will present no phenotypic abnormality, e.g., in one case, whole-body XX/XY chimerism was detected for a mother of two children pregnant with the third, at the age of 46 [3]. Microchimerism is more common, where a mother’s stem cells reach the embryo through the placenta [4] and can be detected well into adulthood. Apart from genetic effects, environmental factors including mental and social constructs can also lead to the incoherence between sex identity from physical features. In 1950s, the distinction between sex and gender terms were introduced, with sex referring to physical characteristics, while gender referring to the psychological make-up and conduct of individuals [5]. Accordingly, the World Health Organization (WHO) defines the term sex that describe the biological and physiological characteristics, while gender is defined as socially defined roles, behaviors, activities, and characteristics that are acceptable in a specific community for men and women. If an individual’s gender and sex are mismatched, they are defined as transgender. Gender is, on the one hand, thought to be a social construct, and on the other, inherent to children, who might realize their own gender between 3–5 years old. Endogenous biology and exposure to prenatal androgens are related to the origin of gender identity. However, no particular genetic locus or region of the brain has ever been reliably established as the sole cause of a transgender identity. There is no evidence to support an exogenous theory for how gender identity develops, despite the possibility that the environment influences development of gender identity [6]. The terms sex and gender are, nevertheless, used interchangeably in everyday life. In scientific research, in studies on the effects of sex hormones (progesterone, estrogen, and estradiol) or genetics on mental illnesses in animals, the term *sex* is frequently used. In human studies, both sex (biological) and gender (environment and experience) are used [7]. Understanding the impossibility of segregating sex and gender aspects of many human traits, the term sex/gender was introduced as “persons/identities and/or aspects of women, men, and people that relate to identity and/or cannot really be sourced specifically to sex or gender” [8].

**Figure 1:**
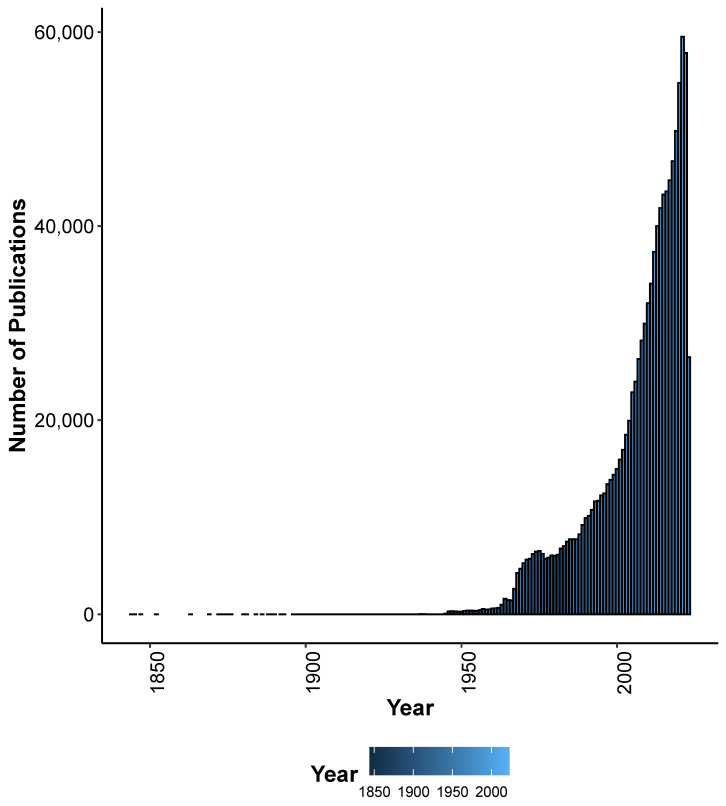
**Number of publications in PubMed after the query ’sex and gender’ as of June 2023.** The bar plot represents the number of publications from the PubMed query, organized chronologically by year of publication.

Many SG differences are present in adult humans, including the endocrine system, gonadal differentiation, reproductive organs, breast differentiation, height, body fat and hair distribution, muscle mass, and density and brain volume and structure. Some of the physiological differences are purely sex differences, i.e., driven by the chromosomes and hormones, such as reproductive organs and gonadal differentiation. Males are often taller, have stronger bones, have more muscle mass and strength, and have higher aerobic capacities. During endurance exercise, females show less muscle fatigue and quicker recovery [9]. Other traits, such as brain structure differences, are considered mostly to be driven by both sex and gender. There is now increasing awareness for taking into account the SG differences in research and strategy. One of the four main objectives of the WHO gender strategy document are to provide qualitative and quantitative information on the influence of gender on health and health care. Accordingly, research has been gathering momentum to collect and analyze sex-stratified data to inform and improve health policies and programs. For example, the ’sex and gender’ query on PubMed resulted in more than 1 million results (performed in June 2023), with an upwards trend, especially in recent decades (Figure 1).

### 1.1. The Evolution of Sex

Sexual reproduction is common in nature, with over 99% of eukaryotes reproducing sexually. Moreover, sexual reproduction is very primitive, estimated to have originated about 2 billion years ago, found even in single cell organisms—protists. Sexual reproduction was selected through evolution to generate variation in a finite population in a changing environment. A known theory in the field of evolution of sex states that sexual reproduction is not beneficial, at least on a superficial level, compared to asexual and hermaphrodite reproductions, since it decreases the number of possible variations in a population [10,11]. However, since most complex species have all adapted sexual reproduction, there must be another benefit than just the number of possible variations of phenotypes. From these and other observations, Geodakyan hypothesized the evolutionary theory of asymmetry, which states that one sex has a tendency of being conservative (females), while the other sex (males) is more responsible for the phenotype variations [10,11]. While this theory can explain some sex-typical behaviors, such as the most power-seeking and risk-taking sex in many species also being the one most vulnerable to cognitive and neurological disorders, it does not explain everything. Moreover, a study conducted in three different cultures (Canada, Russia, and China) highlighted that men, while seeking power and prestige, were less fit for supervisory work [12], questioning sex-specific selection of these traits towards species fitness. Thus, the relationship between the evolution of sex and sex-typical behaviors is not yet fully understood.

As aforementioned, sexual reproduction increases the diversity in the genome of the offspring, which can have benefits to reduce infection risk, e.g., immune gene recombination, response to change in environment, buffer against recessive mutations [13]. In vertebrates, sperms are generated in the thousands—providing genetic diversity at a very low cost, as each sperm is very small. On the other hand, an egg has to be big to provide nutrition, and therefore, the number of eggs is restricted. In most species, females invest in the offspring, likely the source of the vast majority of sex differences between species. Such processes can influence not only physical traits, but also behavior and psychological aspects. Many sexually dimorphic traits were thought to be evolutionary selected through male–male competition. For example, males are usually bigger and have more muscle mass (and more aggression), which has been attributed to male–male competition, though there are other valid theories as well. For example, the sex difference in height previously attributed to males selected for height was challenged by demonstrating that males and females have similar growth trajectories until females stop growing around the age 13, mainly due to reaching puberty earlier through the effect of the hormone estrogen [14]. Male birds are not physically bigger, but have a larger brain network due to song development. Bird songs are very sensitive to testosterone levels and are a key mechanism to attract females and signal male fitness to other birds. Across many species, sex differences are quite small during development and diverge or become exaggerated as they approach adolescence. During adolescence, differences between males and females in physiology, behavior, and risk for psychopathology are heightened. Importantly, animal characteristics, both physical and behavioral, are varied and plastic, making sex traits fluid and malleable. In humans, sex differences are greater culturally than biologically and both sexes are, in fact, far more alike across most traits than they are different [15].

### 1.2. Sex and Gender during Human Development

Genetically, the only sex difference in humans is the presence/absence of sex chromosomes X and Y, including atypical cases, such as testicular feminization and XXY chromosome variants. The sex-chromosome-driven model of sex differentiation puts zygotic inequality at the top of the hierarchy, which is the primary cause of all sex differences [16]. The genes on the sex chromosomes, together with the epigenetic interactions, drive hormonal cascades, which lead to the sex differences in development and disease [17]. The X chromosome is gene-rich and is enriched for genes associated with brain formation and function [18], while the Y chromosome is gene-poor, haploid, and contains male-specific genetically dominant sex-determining factors, including an important sex determining gene—SRY [19]. At conception, sex determination happens as a result of XX or XY chromosome pairing, which triggers the process of sexual differentiation. A very early sex chromosome effect is the expression of XIST, a non-coding RNA expressed from X chromosome to silence parts of one X chromosome in females. The biggest and most primitive sex differentiated event, i.e., X chromosome silencing, is solely designed to reduce the sex differences between males and females.

Gonads define sex at birth and gender identity usually aligns with the sex identity. However, gender identity emerges as a confluence of biological traits, developmental influences, and environmental conditions. The SG differences that are evident early in life prior to socialization are more likely to be biological, as later life differences will be influenced largely by socialization. Indeed, sex differences in humans originate as early as preimplantation. Male embryos has higher metabolic rate than females and prenatal development is slower in males than females, where differences are evident as early as E11.5 [20]. Further during the development, the differences expand; fetuses at 20–22 week gestation showed significant sex differences [21]. These differences further widen after birth. There are small differences at birth, e.g., infant girls make and maintain eye contact more than boys, which get exaggerated in adult life, i.e., sex-related attention biases toward processing object characteristics versus object position location [22]. Many functions develop earlier in female brains than males during development. Males show slower development of language as well as some other visual functions, such as object detection and recognition, and memory functions, such as working and episodic memory [23]. Children usually become aware of the SG differences at about two years of age. By three years, they develop a gender identity, and by four years of age, they are able to identify with a particular gender. At the age of three, children begin to segregate and spend most of their time with the same sex; learning how to work, compete, strengthening the SG biases and most children play with children of the same gender by the age of six [24]. Gender dystopia is thought to be a result of decoupling of timing of sexual differentiation of the brain with the sexual differentiation of the genitals [25].

## 2. Sex and Gender Physiological Differences in the Human Brain

Biological sex differences are largely orchestrated in endocrine and metabolic function. Although less apparent, the brain also undergoes sexual differentiation during development, which results in sex differences, not sex dimorphisms. In utero exposure to testosterone produced by the fetal testis is thought to be the driving factor for divergent brain development in males and females. Early testosterone exposure affects outcomes, including gender identity, sexual orientation, and children’s play behavior [26]. Epigenetic alterations also influence gene expression, which aids in the development of the brain’s sexual differentiation [27]. The two recognized epigenetic processes causing SG differences are genomic imprinting and the inactivation of one of the two X chromosomes in females. Permanent changes in brain structure may result from transient sex differences in gene expression in growing brains, but they may also be prevented by offsetting any possible differentiating effects of sex differences in gonadal hormone levels and sex chromosomal gene expression [28]. Heritability studies suggest that genetic components may play a role, but specific genes have not been identified as of yet [29].

Three main areas are being explored related to the physiological and behavioral outcomes of SG differences in brain development: brain nuclei, neural cell communication, and the communication between the brain hemispheres [27]. Sex differences in brain tissue micro-structure suggest a lesser vulnerability to age-related changes in women [30]. Male brains are consistently bigger than female brains through development, stabilizing at about 10 percent bigger than females. A machine-learning approach revealed the trend of increasing sex difference with age with a difference effect size (d = 1.2) during childhood, which increased further to reach d = 1.6 at age 17 [31]. Careful analysis of sex differences in gray matter volume shows that when the differences are corrected for the total intracranial volume, nearly no sex differences remain statistically significant [32]. Thus, only sex differences in higher white/gray matter ratio, intra- versus inter-hemispheric connectivity, and regional cortical and subcortical volumes hold true as size-independent sex differences [33]. For example, structural and lateralization differences are present independent of size, and SG explains only about 1 percent of the total variance [34]. Despite men having shown to have higher temporal cortex synapse density than women, no sex difference of cerebral energy turnover was observed, demonstrating likely compensatory or countering mechanisms to reduce sex difference [35].

### 2.1. Sex and Gender Differences in Brain Activity and Function

**Figure 2:**
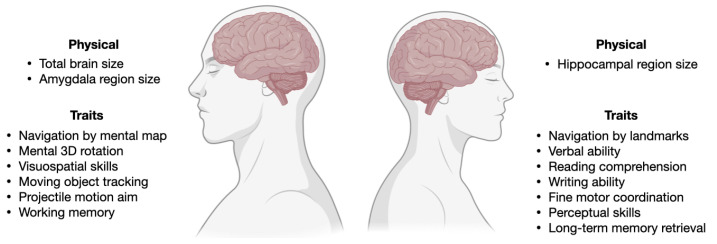
**Figure summarizing SG differences or biases in brain structure and cognitive processes discussed in Section 2**, with examples of the anatomical and physiological differences reported in the literature between male and female brains. The brain is not sexually dimorphic, it must be noted. Thus, rather than distinct features such as gonadal organs, all the traits listed represent mean variations in phenotypes. Created with Biorender.com.

Here, we summarize the scientific literature on SG differences in various brain activities (Figure 2).

**Brain activity:** There are substantial SG differences in brain activity (male > female) in the lateral prefrontal cortex, visual processing regions, parahippocampal cortex, and the cerebellum during long-term memory retrieval [36]. The metabolic connectivity in elderly brains suggests greater efficiency in the posterior default mode network for males, and in the anterior frontal executive network for females [37].

**Executive functions:** There is evidence for SG differences in the neural networks underlying nearly all executive control tasks, according to a systematic literature review of functional neuroimaging studies investigating SG differences in the three important executive control domains: cognitive set-shifting, performance monitoring, and response inhibition [38].

**Memory:** Gender-related brain networks during verbal Sternberg tasks were examined using near-infrared spectroscopy (NIRS) and electro-encephalography (EEG). According to NIRS findings, women outperform men in verbal working memory in terms of both brain activation and connectivity. Men tend to encode memories using a more visuospatial method than women do, according to an EEG (effective connectivity and event-related spectral power) study [39] (Figure 2). A meta-analysis of brain region activation during long-term memory retrieval revealed SG differences (male > female) in the lateral prefrontal cortex, visual processing regions, para-hippocampal cortex, and the cerebellum [36]. Episodic and semantic autobiographical memory also display diverse SG differences [40].

**Language and communication:** SG differences in language are negligible at a population level, but there is an enormous gap in the language deficits. Males are twice as likely than females to fall in the lowest 10th percentile in language tests, and are more often diagnosed with developmental disorders, which rely on tests of language development [41]. From a very young age, girls excel in their ability to read facial expressions, language fluency, and navigating through other social clues. Though the SG differences in each individual trait are small, they add together, making an average girl much better at communication than an average boy (Figure 2).

**Intelligence:** Male brains are indisputably larger then female brains, but this has no effect on men’s and women’s average intelligence [42]. The brain mechanisms that support intelligence involve a network of interconnected regions, including the prefrontal-parietal and basal ganglia, and the network architecture varies between sex and genders [43].

**Emotion:** A study noted that girls had a higher empathy quotient (*p* < 0.05), while boys showed a slightly higher systematizing quotient than girls [44]. Men and women differ in reward-related brain activation, with men showing higher sensitivity to reward and neural sensitivity to both wins, large or small, and losses than women [45]. The pattern of brain activity during the perception of one’s own body in comparison to a jumbled control image did not differ between men and women. Men showed noticeably stronger activation in attention-related and reward-related brain regions when viewing images of other bodies of the same sex or the opposite sex, whereas women engaged stronger activation in striatal, medial-prefrontal, and insular cortices when viewing their own body compared to other images of the opposite sex [46].

In summary, SG differences are noted in many behavioral traits. It is important to note that the data are not homogeneous and consistent about SG differences in many of the abovementioned studies. The differences are small, often with overlapping performances. Furthermore, other variables can play a role, such as ethnicity. The largest baseline differences, after correcting for age and education, were between non-Hispanic white women and black men on memory, and between non-Hispanic white men and Hispanic women on visuospatial and language skills. Memory and visuospatial decline varied across racial/ethnic groups, with black women seeing sharper declines in memory and visuospatial abilities than Hispanic males and non-Hispanic white women, respectively [47]. Additional longitudinal studies with extremely large multicultural samples stratified in distinct, well-sized, and precise age groups, taking into account biological and sociocultural characteristics, are required given the involvement of a number of variables and the interactions between them [48].

### 2.2. Structure Function Correlates and Causal Factors

Despite some evidence of SG biases in learning and intelligence, and consequently, behavior, the structure function correlates of them are largely missing. SG differences in hemispheric asymmetry are certainly not the driving force behind SG differences in cognitive functioning [49]. Task-based functional magnetic resonance imaging (fMRI) failed to find reproducible activation differences between men and women in verbal, spatial, or emotion processing [34]. Handful examples of likely structure-function correlates are described below.

- Models of brain gray matter volume and concentration might distinguish between men and women with higher than 93% accuracy [50]. Significant associations with gray matter volume were detected for neuroticism, extroversion, and conscientiousness only in males [51]. Gray matter morphology in the brain, as determined by magnetic resonance imaging (MRI), and self-reported psycho-social traits are found to be associated with these traits differently in men and women [52].
- The endocannabinoid system, which includes the cannabinoid CB1 receptor (CB1R), is crucial for the development of the brain, cortical rhythms, plasticity, reward, and stress sensitivity. There was a significant difference in the CB1R SG, and working memory and CB1R availability were both correlated [53].

Non-binary SG-related factors, such as age, education, socioeconomic status, self-esteem, sexual identity, and orientation, might explain individual differences better than sex or gender [54,55]. SG should, therefore, be considered as an imperfect proxy of a combination of yet-unknown biological and psycho-social factors underlying SG differences [56].

## 3. Sex and Gender Differences in Psychopathology

**Figure 3:**
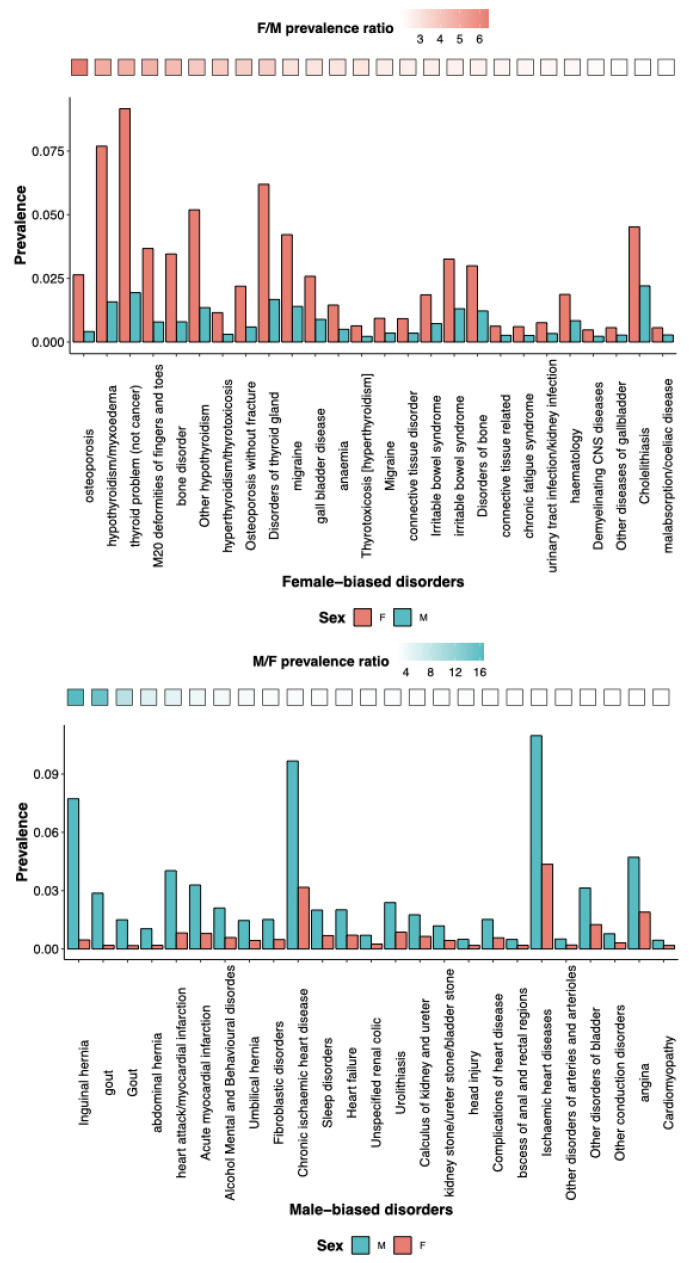
**Self-reported skewed prevalence of disorders between the sexes.** (**top**) Female-biased and (**bottom**) male-biased prevalence of disorders as reported by individuals from the UK Biobank data. In each plot, the disorders are ordered based on the highest ratio of prevalence between the two sexes, represented by the gradient at the top of each bar plot.

Other reviews have discussed in detail sex and gender in neurological pathologies, both from an evolutionary [57] and from a clinical management perspectives [58]. Here, we further elaborate on SG differences in specific mental health disorders, mostly based on sex-biased prevalence. SG differences result into sex-specific vulnerabilities, resulting in biases in disease prevalence. The self-reported disorders from UK bio-bank data show that females carry a disproportionate burden of thyroid problems and bone and immune disorders, while males carry a disproportionate burden of diabetes and cardiovascular and sleep disorders (Figure 3). Sexually selected traits can bring vulnerabilities, i.e., sensitivity to stressors, and can generate SG disparities in health. Premature baby girls show a higher language deficit than premature boys, and boys exposed to prenatal toxins show greater spatial skill deficiency. Many common vulnerabilities, such as poor nutrition, chemotherapy side effects, anorexia, and alcohol abuse, will, therefore, have an SG component [59], including in psychopathology. For instance, those who scored higher on the male-biased differentiation scale had higher levels of male-biased psychopathology (externalizing symptoms, such as disruptive behaviors), while those who scored higher on the female-biased differentiation scale had higher levels of female-biased psychopathology [60]. The genome has an impact on SG disparities as well. The region comprising NKAIN2, which interacts with sodium/potassium-transporting ATPase (adenosine triphosphatase) enzymes and implicates neuronal excitability, was found to have genome-wide significant single nucleotide polymorphism-by-sex interaction across mental illnesses. Gene-based analyses identified a G × S interaction across disorders with transcriptional inhibitor SLTM [61]. The disparities in mental problems between the SGs can be attributed to both environmental and psycho-social factors. Notably, studies show that risk factors such as discrimination, domestic violence, and sexual abuse can also contribute to the increased prevalence of mental illness in women. Gender inequalities in mental disorders are mostly a result of the substantially higher occurrence of childhood sexual and emotional abuse in women than in men [62].

**Figure 4:**
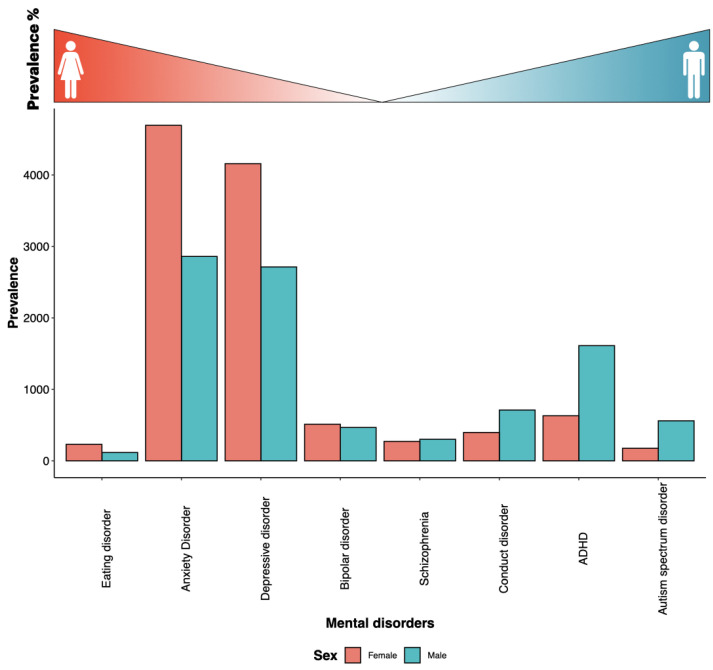
**Sex-biased prevalence of mental disorders.** Global age-standardized fraction of males and females from each mental health disorder from the global mental health prevalence statistic 2019 [65]. The disorders are arranged female-biased to male-biased prevalence, from left to right, as indicated by the gradient at the top of the bar plot.

Most medical diagnoses present somewhat differently in men and women, moreso at specific periods of life (Figure 4). Women are more vulnerable to psycho-social environmental stressors (due to sex hormone influence and blunted hypothalamo–pituitary–adrenocortical axis (HPA axis) stress responses), leading to a higher prevalence of mental disorders. With the exception of late-onset schizophrenia, women have significantly higher chronic prevalence of anxiety, depressive, and bipolar disorders [7]. There are nearly twice as many women as men suffering from Alzheimer’s disease (AD) and major depression (Figure 4). Additionally, antipsychotic medications work at lower levels on women’s psychotic symptoms than they do on men’s. This implies that a lot of women might overdose and suffer needless side effects as a result [63]. Letrozole, an aromatase inhibitor, is being researched in preclinical models as a potential treatment for high-grade gliomas, because this cancer frequently expresses estrogen synthase aromatase (CYP19A1). Female rats had much slower letrozole clearance, which led to noticeably greater plasma and brain drug concentrations [64]. On the other hand, autism is a diverse group of early-onset neurodevelopmental disorders that affects more men than women (Figure 4).

### 3.1. Anxiety, Depression, and Stress

Clinical anxiety and depression are chronic conditions of altered mood regulation and the most prevalent among mental illnesses. They carry a large societal burden, including around 15% mortality due to suicide, and have striking sex or gender differences in onset, incidence, and severity of these conditions. Women have twice the rate of depression and anxiety disorders as men and are four times as likely to have recurrent conditions (Figure 4). Women show greater sensitivity to negative emotions or responses to fear, threat, loss, and frustrating non-reward. For example, Labonte et al. found limited overlap between transcriptional patterns in major depressive disorder (MDD) between SG and identified key regulators of sex-specific gene networks underlying MDD, including boosting ERK signaling and pyramidal neuron excitability, and downregulation of the female-specific hub gene Dusp6 in the rodent prefrontal cortex, which replicated stress vulnerability in females but not males [66]. Intriguingly, there was no change in SG on the memory of words associated with occupational stress, either before or after exposure to stress. Therefore, it does not appear that sex-specific cognitive bias is a component in explaining disparities between healthy male and female employees in the SG of stress-related mental health illnesses [67]. Thus, SG differences in emotion regulation are noted, but their neural basis remains poorly understood. Women may use a frontal top–down control network to downregulate negative emotion due to their tendency for stronger emotional reactivity, but men may use posterior parts of the ventral attention network to divert attention away from the negative input [68]. A recent neural correlate study found that greater brain dynamism was positively correlated with anxiety and depression traits in males, while it was positively correlated with drive, novelty-seeking, and self-control in females. These neural correlates of valence, anxiety, and depression traits are significantly different in males and females [69]. Another study noted that gender role is an important determinant in the interpretation of SG differences in emotional reactivity [70] and gender roles (not sex) shape the susceptibility to emotion [71]. Interestingly, a recent neuroimaging study, which included a transgender group as well, found a major effect of sex on gray matter volume irrespective of the self-identification as a woman or man. The neuroanatomical signature of sex in cisgender individuals did not interact with the female depressive traits [72].

### 3.2. Aggression and ADHD

Childhood aggression brings enormous costs to families and societies and it often accompanies other problems. Typically, more boys than girls are affected by aggression and ADHD. A recent multi-cohort study replicated SG differences in average aggression scores at most ages, with a correlation of 0.5 between aggression and ADHD-related problems [73]. Boys with ADHD showed smaller accumbens, amygdala, and hippocampal sizes than boys with usual development. There were no volumetric differences between girls with ADHD and girls who were usually developing in any structure [74].

### 3.3. Alcohol

Though males in general have higher susceptibility, progression, and clinical outcomes of alcohol dependence than females, over the past decade, a much higher increase of alcohol use disorder has been noted in females (84%) than males (35%). Alcohol consumption for emotion regulation may differ between men and women, as may the brain responses. Both chronic ethanol exposure and binge drinking exhibit neuroimmune markers that are SG-dependent [75]. Alcoholic men’s brain activation in areas such as the middle and superior frontal cortex, the precentral gyrus, and the inferior parietal cortex was significantly lower than that of nonalcoholic men’s, whereas alcoholic women’s brain activation in areas such as the superior frontal and supramarginal cortex was higher. Alcohol, thus, boosted brain activity in women while decreasing it in men [76].

### 3.4. Trauma

Significant SG differences were observed in all pediatric ages, including neonates/infants, prepubertal children, and adolescents, in both the response to and recovery from traumatic brain injury [77]. Women are twice as likely than men to suffer post-traumatic stress disorder (PTSD), which affects about 10% of them in their lifetime. There is a highly connected, downregulated set of interneuron transcripts in PTSD prefrontal cortex. Interneuron gene ELFN1 confers a significant genetic liability and a likely functional role in PTSD pathophysiology specifically in females [78].

### 3.5. Autism

Autism is a male-biased disorder with significantly higher lifetime prevalence in males than females. This leads to misdiagnosis and missed diagnosis for many females with an autism spectrum disorder. There are greater differences between typically developing children and those with autism spectrum disorder in females than in males [79]. Typical cross-hemispheric interactions involved in autism might originate from sex-dependent factors. A study of SG differences in autism spectrum disorder-related alterations in brain asymmetry found greater changes in females with autism compared with males with autism, revealing a “female-protective effect” [80], which combined with a “female camouflage effect” [81] can lead to underdiagnosis of female autistic patients. Additionally, it was discovered that there were no SG differences in sensorimotor performance between ordinarily developing men and women, and that autistic women were more likely than autistic males to experience sensorimotor symptoms [82].

### 3.6. Seizure

Men are typically more prone to excitability episodes and seizure activity than women, but it is unclear what molecular factors cause these disparities. It was discovered that the vulnerability of men and women to seizures and epileptogenic cascades varied depending on regional morphology and neural circuits [83]. Disparities in brain development, neurogenesis, neuronal chloride homeostasis, and neurotrophic and glial responses are potential neurobiological grounds for SG disparities in epilepsy [84].

### 3.7. Neurodegenerative Disorders

Numerous factors, including genetics, lifestyle choices, and other medical problems, affect the symptoms of neurodegenerative illnesses. Age progression is by far the greatest risk factor. Additionally, SG play a role in the development of neurodegenerative disorders, such as Alzheimer’s, Parkinson’s, Huntington’s, and multiple sclerosis, with considerable differences in disease prevalence and severity between the sexes [85]. Age and SG influence each other, i.e., the metabolic makeup of the brain and its relative rise in activity and adaptability with time may actually increase both vulnerability to and resistance to neurodegenerative illness [86]. When compared to the male brain, the metabolic brain age of women is consistently lower than that of men throughout their adult lives [87].

#### Alzheimer’s Disease

The lifelong risk of AD is twice as high in women as it is in males, and the incidence rates for women are higher in low- and middle-income nations. There are numerous possibilities, including the lower educational attainment of women, the survival bias against men, and genetic and hormonal causes, but no conclusive research have been performed so far. Cognitive deterioration occurs at higher rates in women with AD. According to several research, women with AD exhibit higher rates of behavioral signs and dependency. In addition to dying sooner than women with AD, men also exhibit higher cognitive impairment. On the other hand, females with mild cognitive impairment exhibited more neurodegeneration and quicker decline than males did, and early tau deposition was higher in women than in men in those on the AD trajectory [88]. Greater understanding of these differences will improve outcomes for AD diagnosis and treatment for both SGs [89]. For example, men have been found to benefit from cholinesterase inhibitor therapy for AD in a greater and more focused way [90]. Deviations in brain structure and biomarkers, psychosocial stress responses, pregnancy, menopause, sex hormones, genetic background (i.e., APOE), inflammation, gliosis, immunological module (i.e., TREM2), and vascular illnesses are the key SG-biased risk factors for AD [91]. Although some of the SG differences in AD prevalence are due to differences in longevity, other distinct biological mechanisms increase the risk and progression of AD in women. The X chromosome affects AD-related vulnerability in mice expressing the human amyloid precursor protein (hAPP), a model of AD. A second X chromosome conferred resilience potentially through the candidate gene KDM6A, which does not undergo X-linked inactivation [92]. The expression levels of CHI3L1 were correlated with age and gender. Female brains showed higher CHI3L1 expression than male brains. The expression differences between men and women were most obvious in older subjects. The expression analysis of CHI3L1 in the different brain regions of AD subjects also showed SG differences [93].

### 3.8. Schizophrenia

Schizophrenia has been considered a disorder of young men. The epidemiology shows a slight male bias in disease prevalence (Figure 4). SG differences in schizophrenic patients’ transcriptomes showed enrichment for molecular pathways related to epigenome regulation, synaptic transmission, and hormone regulation; furthermore, gene expression in schizophrenia was less affected in females compared to males [94]. For the majority of cognitive tests, age-related changes in the structure and function of the brain in schizophrenia were similar in men and women [95]. In a large sample of schizophrenia patients after adjusting for sex, age of onset, severity of condition, and education, gender was linked to the presence of depression. Therefore, using gender as a representative personality attribute rather than sex may provide valuable insights on how schizophrenia manifests [96].

### 3.9. Other Disorders

Women display significantly higher incidence of pain disorders [97], and numerous underlying mechanisms, including pain management, have been researched, including behavioral and biological factors [98]. The clinical and epidemiological features of bipolar disorder also display SG differences, such as in the dysfunctions of the cortico-limbic neural system in bipolar disorder [99]. Furthermore, the physical health of a person is affected by one’s psychological well-being. SG differences in brain, therefore, influence SG divergence in other non-communicable diseases. Epidemiological studies have established strong links between cardiovascular and several psychological conditions, including depression, chronic psychological stress, post-traumatic stress disorder, and anxiety [100]. Higher type 2 diabetes mellitus prevalence were observed in women with severe mental illness compared to men [101]. Although the prevalence of obesity has increased globally over the past 40 years in both men and women, women continuously experience higher rates of obesity than males. All over the brain, obesity has been linked to structural, functional, and chemical changes. Men’s obesity appears to be correlated with changes in the somatosensory system, whereas women’s obesity appears to be more correlated with changes in the reward system. SG variations have also been seen in the brain response to taste in obese individuals [102]. There have also been reports of SG variations in eating habits and food perception. Compared to males, females activated the frontal, limbic, and striatal brain regions, as well as the fusiform gyrus more when exposed to visual food signals [103].

## 4. Sex and Gender Differences at Cell and Tissue Level

The advent of microarray technologies allowed genome-wide characterization of gene expression at a tissue level. Ref. [104] studied SG-specific gene expression in a post-mortem human brain using microarray and identified six differential expressed genes between male and female on sex chromosomes (DBY, SMCY, UTY, RPS4Y, and USP9Y on the Y chromosome and XIST on the X chromosome). The advances of RNA sequencing (RNA-seq) technologies together with large consortia initiatives allowed speed up characterization of SG differences across many tissues and cell types. Ref. [105] studied the sex differential transcriptome across 53 human tissues using RNA-sequencing data from the Genotype-Tissue Expression (GTEx) project (544 adults, v.6, Refs. [106,107]) and a population variation data from the 1000 Genomes Project [108]. They found the SG differential gene expression in 45 common tissues. SG-biased expression varied greatly among tissues, especially the sexual dimorphic ones, such as mammary glands (female-biased expression) and testis (male-biased expression). Male-biased genes were more common in the skin, skeletal muscle, and cingulate cortex tissues, while female-biased genes were more common in the liver, heart, skin, skeletal muscle, and a group of mostly X-liked genes. Female-biased genes were associated with obesity, muscular diseases, and cardiomyopathy. They noted no significant differences in age differences between males and females. Ref. [109] analyzed and identified female-biased and male-biased genes across 14 different healthy tissues from GEO and GTEx databases. SG-biased genes were enriched more for sex chromosomes than autosomes across tissues. However, globally, 90 percent of SG-biased genes were mapped to autosomes. Male-biased genes were greater in number and more shared across tissues than female-biased genes. An evolutionary analysis showed male-biased genes have slower evolutionary rates, higher homologous gene numbers, and an earlier origin in phyletic evolution. Ref. [110] studied the biological mechanisms for tissue-specific sex differences (TSSD) across 40 tissue types in GTEx (v.7) data and identified 65 autosomal and 66 X-linked TSSD transcripts. They noted X-linked KAL1 gene for TSSD in gene expression, with higher expression in females than males in lung tissue. This is consistent with [111], showing bi-allelic expression of KAL1 gene in lung tissue, which provided evidence for tissue-specific escape from X-activation. Similar distances between the closest androgen and estrogen binding motifs and enhancer from the cis-expression quantitative trait loci (eQTLs) of TSSD suggested that the SG-differential expression may be influenced by the androgen and estrogen regulatory components in a cis region.

### 4.1. Spatio-Temporal Patterns in Specific Brain Regions

Ref. [112] studied SG differences in gene expression using post-mortem adult brain and spinal cord samples. In total, 2.6% (448/17,501) of all genes in human central nervous system (CNS) showed SG-differential expression. Sex-biased genes were present on both sex-chromosome and autosomes. Ref. [113] examined the transcriptome in human prefrontal cortex from age 1 month to 50 years, and found that 83 genes (25 on sex chromosomes and 58 on autosomes) with differential expression between males and females. Ref. [114] also analyzed SG differences in 11 brain regions of healthy adults using bulk RNA-sequencing. In their analysis, SG-biased genes were enriched for Y chromosome in males and X chromosome in females, as previously described. Female-biased genes were enriched for synaptic membrane and lumen, and male-biased genes for mitotic processes. Most of the SG-biased genes were expressing androgen, but not estrogen, response element binding sites, indicating a possible role in the regulation of these genes by testosterone. However, they also found that age, more than sex, affected gene expression. A recent study observed that differences between SGs in physical and verbal skills, while present in younger years, tended to decline with age. This phenomenon was defined by the authors as “middle age–middle sex” [115], which highlights the importance of not only focusing on SG but also on age when studying such differences.

Ref. [116] studied the puberty-associated gene in male and female mice and humans. They found that over 40 puberty-associated genes in the pituitary gland showed SG-biased gene expression. In childhood, more brain regions have female-biased genes and puberty stage showed the dominance of male-biased genes. No such trend was observed in adulthood. Ref. [117] analyzed brain tissue at different ages in healthy mice and humans and found that a ‘genetic lifespan calendar’ controlled every cell type in the brain. The peak of gene expression reorganization occurred around 26 years of age in humans, and the genes affected included those associated with schizophrenia and synaptic-related (PSD, PSD95 complex) genes. Women showed a slightly delayed calendar of changes compared with men (26 years for males and 27.5 for females), which was also conserved in mice. Ref. [118] studied the spatio-temporal dynamics of the human brain transcriptome by examining 16 brain regions from embryonic development to late adulthood. Using PCA and MDS techniques, it was discovered that region and age contribute more to the overall differences in gene expression than do other factors (sex, ethnicity). They also discovered that exon usage varied among regions, ages, and both in 90% of expressed genes. Ref. [119] generated human fetal brain expression data and found that the Y-chromosome genes had the highest SG differences, indicating that there is a prenatal SG bias in brain expression. All of the brain’s regions showed the presence of 10 of the 11 Y-chromosome-encoded genes (RPS4Y1, PCDH11Y, DDX3Y, USP9Y, NLGN4Y, EIF1AY, UTY, ZFY, TMSB4Y, CYorf15B, and PRKY). In the human fetal brain, more than one-third of the genes were on the Y chromosome, demonstrating their significance for the formation of the SG-biased brain, Ref. [120] studied the transcription profile of four developmental stages (prenatal, early childhood, puberty, and adulthood) in more than 14 regions of human brains using RNA-seq data. Male-biased genes were highly enriched for neurological and psychiatric disorders (autism, bipolar disorder, schizophrenia, AD, and Parkinson’s disease), while female-biased genes were barely significant enriched for a few diseases (OCD, AD, schizophrenia, and epilepsy), which suggested that the male-biased genes likely have functional consequences relevant to human brain diseases, consistent with the “female protective model” in neurodevelopmental disorder. In our latest study [121], we analyzed publicly available single-nucleus RNA-sequencing datasets of the human cortex, spanning from the second trimester of gestation until geriatric age, and including both healthy individuals and patients suffering from AD and MS. Female-biased genes mainly enriched for brain-related processes, while male-biased genes enriched for metabolic pathways. We also found a female-biased upregulation of mitochondrial genes in neuronal populations in most of the datasets, indicating a potential source of the previously described difference in metabolism [122]. Most SG-biased genes, both in females and males, are consistent in all cell types and developmental stages, suggesting androgens as potential key regulators of SG bias.

### 4.2. Brain Pathologies

Ref. [123] reported gene expression difference between AD and healthy subjects considering the factors of age, sex, and tissue and identified 46 differential expressed genes (DEGs) with differential regulation between males and females. Chemokine receptor type 4 (CXCR4) among these genes showed a statistically significant pairwise interaction between sex and illness status. Both in AD and in females, CXCR4 was upregulated. Ref. [124] reported a meta-analysis of SG effects on AD gene expression. They identified 1903 DEGs in male and 2333 DEGs in female (1640 genes were female specific) in AD, where female-specific genes were involved in pathways associated with neurodegenerative diseases, such as oxidative phosphorylation, AD, Huntington’s disease, and Parkinson’s disease pathways. Ref. [66] studied a combination of differential expression and gene co-expression network analyses to characterize the sexual dimorphism of major depressive disorder (MDD) of six brain regions. Ref. [125] sequenced 3589 cells from both the tumor core and the peritumoral brain using single-cell RNA analysis on a cohort of four patients to study glioblastoma.

## 5. Concluding Remarks and Future Perspectives

In summary, age, financial position, education, sexual orientation and identity, gender roles, sex hormones, and others all have an impact on SG differences in the brain [54]. As there is not a single distinguishing feature of a male or a female brain, the framework of a male–female continuum needs to be replaced with mosaic brains residing in a multidimensional space [126]. Finally, the findings of SG differences in brain development and aging may depend on the analysis (e.g., quantitative versus topographic), the data (e.g., structural versus metabolic, or cohort effects), and one’s point of view (e.g., inferential statistics versus predictive machine learning) [86]). Furthermore, brains are plastic during development and lifespan. London taxi drivers have larger posterior hippocampi and hippocampal volume correlated with the amount of time spent as a taxi driver [127]. A study of transgender males and females explored hypothalamus and noted that while transgender female-to-males individuals in their sample were similar to cisgender male individuals in the control sample, transgender male-to-female individuals were similar to control cisgender female individuals. These hypothalamic regions were, therefore, more closely linked to gender identity than to chromosomal sex [5]. Importantly, gender differences include societal attitudes and prejudices reflected in individual behaviors, likely influencing the sex differences. For example, a new study found that younger males reported higher self-estimated intelligence than females, and this pattern was reversed in older age. Furthermore, self-estimated intelligence could be significantly predicted by age, sex, physical attractiveness, and self-estimated emotional intelligence [55].

Big data generated by emerging technologies and integrative analyses of data offer new opportunity to eliminate SG gaps in health outcomes. This will include establishment of sex-stratified clinical decision support systems. For example, acute myocardial infarction in women may go undiagnosed due to the overall clinical decision limitations, as females had substantially lower upper reference limits of hs-cTnI and hs-cTnT than overall clinical decision limits of 26 ng/L and 14 ng/L [128]. Development of novel data analysis approaches integrating multi-omics data (e.g., genetics, eQTL, mQTL, and pQTL), together with other clinical and demographic factors, such as hormonal status, education, and socio-economic factors, is, therefore, needed, which specifically performs SG-stratified and SG-interaction analysis of all data (in addition to SG-adjusted analysis). The establishment and improvement of longitudinal cohorts with repeated assessments of clinical, cognitive, and biomarker variables, such as peripheral (blood, saliva, and cerebrospinal fluid), multi-omics (transcriptome, epigenome, proteome, and metabolome), and genetic data, are, thus, necessary for the systematic identification of SG differences. Importantly, the cohorts must target recruitment at specified age ranges, including peri- and post-menopausal women, age-matched men, and underrepresented communities. The continuous improvement in diagnosis, prognosis, and therapy of diseases is accelerated as a result of technology advancements (such as omics and wearables) [129]. For the benefit of society, there is an urgent need for the systematic integration of these technologies into healthcare and national healthcare systems.

## Data Availability

Not applicable.

## Abbreviations

The following abbreviations are used in this manuscript:

SG	Sex and gender
CB1R	Cannabinoid CB1 receptor
AD	Alzheimer’s disease
OR	Odds ratio
CYP19A1	Estrogen synthase aromatase
MDD	Major depressive disorder
PTSD	Post-traumatic stress disorder
hAPP	Human amyloid precursor protein
TSSD	Tissue-specific sex differences
QTLs	Quantitative trait loci
CNS	Central nervous system
DEG	Differential expressed genes
CXCR4	Chemokine receptor type 4
GTEx	Genotype-Tissue Expression
hs-cTnI	High-sensitivity cardiac troponin I
hs-cTnT	High-sensitivity cardiac troponin T
